# Metabolic Profiling of Human Eosinophils

**DOI:** 10.3389/fimmu.2018.01404

**Published:** 2018-06-21

**Authors:** Linsey Porter, Nicole Toepfner, Kathleen R. Bashant, Jochen Guck, Margaret Ashcroft, Neda Farahi, Edwin R. Chilvers

**Affiliations:** ^1^Division of Respiratory Medicine, Department of Medicine, University of Cambridge School of Clinical Medicine, Cambridge, United Kingdom; ^2^Biotechnology Center, Center for Molecular and Cellular Bioengineering, Technische Universität Dresden, Dresden, Germany; ^3^Division of Renal Medicine, Department of Medicine, University of Cambridge School of Clinical Medicine, Cambridge, United Kingdom

**Keywords:** eosinophil, neutrophil, oxidative phosphorylation, metabolism, glycolysis, hypoxia, atopy, real-time deformability cytometry

## Abstract

Immune cells face constant changes in their microenvironment, which requires rapid metabolic adaptation. In contrast to neutrophils, which are known to rely near exclusively on glycolysis, the metabolic profile of human eosinophils has not been characterized. Here, we assess the key metabolic parameters of peripheral blood-derived human eosinophils using real-time extracellular flux analysis to measure extracellular acidification rate and oxygen consumption rate, and compare these parameters to human neutrophils. Using this methodology, we demonstrate that eosinophils and neutrophils have a similar glycolytic capacity, albeit with a minimal glycolytic reserve. However, compared to neutrophils, eosinophils exhibit significantly greater basal mitochondrial respiration, ATP-linked respiration, maximum respiratory capacity, and spare respiratory capacity. Of note, the glucose oxidation pathway is also utilized by eosinophils, something not evident in neutrophils. Furthermore, using a colorimetric enzymatic assay, we show that eosinophils have much reduced glycogen stores compared to neutrophils. We also show that physiologically relevant levels of hypoxia (*P*O_2_ 3 kPa), by suppressing oxygen consumption rates, have a profound effect on basal and phorbol–myristate–acetate-stimulated eosinophil and neutrophil metabolism. Finally, we compared the metabolic profile of eosinophils purified from atopic and non-atopic subjects and show that, despite a difference in the activation status of eosinophils derived from atopic subjects, these cells exhibit comparable oxygen consumption rates upon priming with IL-5 and stimulation with fMLP. In summary, our findings show that eosinophils display far greater metabolic flexibility compared to neutrophils, with the potential to use glycolysis, glucose oxidation, and oxidative phosphorylation. This flexibility may allow eosinophils to adapt better to diverse roles in host defense, homeostasis, and immunomodulation.

## Introduction

Eosinophils are innate immune cells that represent approximately 5% of the circulating leukocyte population (1). Traditionally, eosinophils are thought to play a role in host defense against parasites but also play a major role in promoting allergic inflammation (2, 3). Eosinophils fulfill this pro-inflammatory role *via* release of their granule proteins, leukotrienes, chemokines, and cytokines (4). However, newly emerging functions of eosinophils include a broader participation in homeostasis and immunomodulatory roles in the liver, adipose tissue, and the small intestine (5–7). The recent identification of a resident lung eosinophil population (rEos) and a separate allergen induced inflammatory eosinophil population (iEos) indicates that phenotypically distinct eosinophils exist, and that these subsets likely perform differing roles (8).

Crucial to our understanding of eosinophil biology is an insight into their energy and metabolic pathways. The metabolic requirements of immune cells are wide-ranging as these cells need to be able to migrate and adapt their metabolic machinery to mount the required immunological response. It is known that naive T cells can switch their metabolism when exposed to foreign antigens (9), macrophages likewise exhibit notable metabolic plasticity depending on their M1/M2 status (10), and neutrophils are reliant on glycolysis to perform phagocytosis and neutrophil extracellular trap formation (11, 12). While there is extensive literature on the bioenergetic profile of these immune cells, data on eosinophil metabolism remains extremely limited, and there are no investigations on eosinophils in disease (13).

The aim of our work was to assess key metabolic parameters (glycolysis, glucose oxidation, and mitochondrial oxidative phosphorylation) in purified human blood-derived eosinophils and compare their metabolic profile to neutrophils. To assess these parameters, we measured eosinophil and neutrophil extracellular acidification rates (ECAR) and oxygen consumption rates (OCR) in an extracellular flux analyser. This technology allows the real-time assessment of live cell metabolism and has previously been used to assess neutrophil, monocyte, lymphocyte, and platelet bioenergetics (14). Our data suggest that while eosinophils and neutrophils both use glycolysis, eosinophils also access additional pathways such as glucose oxidation and mitochondrial oxidative phosphorylation. This greater metabolic flexibility may allow eosinophils to adapt better to their different tissue locations and perform both pro-inflammatory and homeostatic roles.

## Materials and Methods

### Study Subjects

Human eosinophils and neutrophils were isolated using blood from atopic and non-atopic volunteers. Atopy was defined as volunteers with appropriate history and a positive skin prick test to one or more aeroallergens (the majority of the volunteers tested positive for timothy-grass pollen). The subjects all gave written informed consent and the study was approved by the Cambridgeshire 2 Research Ethics Committee (06/Q0108/281).

### Neutrophil and Eosinophil Isolation

Neutrophils were isolated from human peripheral blood using discontinuous plasma-Percoll gradients as previously described (15). Eosinophils were obtained from human peripheral blood using the RoboSep system (StemCell Technologies, Vancouver, BC, Canada) as described (16). We routinely purify neutrophils to ≥95% purity and eosinophils to ≥98% purity, with viability of both cell types ≥99% (as assessed by trypan blue exclusion).

### Measurement of ECAR and Oxygen Consumption Rate (OCR)

Sensor cartridges (96-well) were hydrated for a minimum period of 12 h with XF Calibrant solution according to manufacturer’s instructions (Seahorse Biosciences, North Billerica, MA, USA). XF Assay Base Media (Seahorse Biosciences) was supplemented with l-glutamine (2 mM) for ECAR measurement or supplemented with l-glutamine (2 mM), sodium pyruvate (2 mM), and glucose (10 mM) for OCR measurement. All media was pH 7.35 ± 0.05 and 0.2-µm sterile filtered prior to use.

Cell culture plates (96-well) (Seahorse Biosciences) were coated with 30 µl of Cell-Tak (Corning) (22.4 µg/ml) and left for a minimum period of 12 h at 4°C. Cell-Tak was removed and wells washed with the appropriate XF Base Media before addition of cells. Granulocytes (3 × 10^6^/ml in XF Assay Base Media) were spun (acceleration 2 and brake 0, 58 *g* for 30 s) onto 96-well plates before incubation in a non-CO_2_ incubator at 37°C for 1 h. Measurement of ECAR and OCR was performed in a 96-well XF Extracellular Flux Analyser (Seahorse Biosciences).

Sensor cartridge injection compounds or XF Base Media controls were injected in a total volume of 25 µl. Injection compound concentrations were as follows: glucose (10 mM), oligomycin (OLIGO) (mitochondrial ATP synthase inhibitor) (2.5 µM), 2-deoxy-d-glucose (2-DG) (100 mM), carbonyl cyanide-4-(trifluoromethoxy)phenylhydrazone (FCCP) (1.5 µM), the mitochondrial complex I inhibitor rotenone (1 µM), glutamine (2 mM), phorbol 12-myristate 13-acetate (PMA) (200 nM), and the mitochondrial complex III inhibitor antimycin A (1 µM) unless otherwise stated in the figure legend.

Glycolysis parameters [glycolysis, glycolytic capacity (GC), glycolytic reserve (GR), and non-glycolytic acidification (NGA)] and oxidative phosphorylation parameters [basal respiration (BR), proton leak, ATP-linked production, maximum respiration capacity, spare respiratory capacity, and non-mitochondrial-derived OCR] were calculated as previously described (17). A summary of these calculations is provided in Table 1 [adapted from literature provided by Agilent Technologies (18)].

**Table 1:** Calculation of glycolytic and oxidative parameters.

Glycolytic parameter	Calculation
Glycolysis (G)	(Maximal rate measurement after glucose injection through measurement prior to oligomycin (OLIGO) injection) minus (measurement prior to glucose injection)
Glycolytic capacity (GC)	(Maximal rate measurement after OLIGO injection through measurement prior to 2-DG injection) minus (measurement prior to glucose injection)
Glycolytic reserve	(GC) minus (glycolysis)
Non-glycolytic acidification (NGA)	Measurement prior to glucose injection

**Oxidative parameter**	**Calculation**

Basal respiration (BR)	(Last measurement prior to OLIGO injection) minus (non-mitochondrial-derived OCR value)
Proton leak (PL)	(Minimum rate measurement after OLIGO injection) minus (non-mitochondrial-derived OCR value)
ATP-linked production (ATP LINK)	(Basal rate) minus (PL)
Maximum respiration capacity (MAX CAP)	(Maximal rate measurement after FCCP injection) minus (non-mitochondrial-derived OCR value)
Non-mitochondrial-derived OCR (NON-MITO)	Minimum rate measurement after addition of rotenone/antimycin A through to the end of the assay
Spare respiratory capacity (SPA CAP)	(Maximum respiration capacity) minus (BR)

*This table is adapted from data provided by Agilent Technologies (18)*.

### ECAR and OCR Measurements Under Hypoxia

The Extracellular Flux Analyser was placed in an *Invivo* 400 hypoxic chamber (Baker Ruskinn, UK) to provide ECAR and OCR measurements under hypoxia (oxygen concentration of 0.8% and 0.1% CO_2_). The Extracellular Flux Analyser, sensor cartridges, XF Base Media, and Cell-Tak coated plates were allowed to equilibrate in the *Invivo* 400 chamber for at least 12 h prior to start of experiment. Granulocytes were harvested as described above, plated onto Cell-Tak coated plates, and incubated in the *Invivo* 400 hypoxic chamber for 1 h prior to OCR and ECAR measurements. A hypoxic environment was confirmed using an ABL5 blood gas analyser (Radiometer, Denmark). Additional sodium sulfite (Sigma-Aldrich, UK) injections (100 mM) were added to provide a 0 oxygen reference and the data analyzed using XF Hypoxia Rate Calculator software (Seahorse Biosciences) according to manufacturer’s instructions.

### Measurement of Reactive Oxygen Species

Granulocytes (3 × 10^6^/ml) were primed with tumor necrosis factor-alpha (TNF-α), 20 ng/ml; GM-CSF 10 ng/ml (R&D Systems, Abingdon, UK), or phosphate-buffered saline (PBS) vehicle control for 30 min and reactive oxygen species generation subsequently measured as peak *N*-formyl-methionyl–leucyl–phenylalanine (100 nM) (fMLP; Sigma-Aldrich, UK)-stimulated, horseradish peroxidase (HRP; Sigma-Aldrich, UK)-catalyzed luminol (Sigma-Aldrich, UK) chemiluminescence as described previously (19).

### Real-Time Deformability Cytometry (RT-DC)

RT-DC measurements were performed to determine the morpho-rheological properties of cells as described previously (20). Eosinophils were resuspended at 2.5 × 10^7^ cells/ml in 1× PBS containing 0.5% methylcellulose to adjust the viscosity to 15 mPas (CellCarrier, Zellmechanik Dresden, Germany). Cell suspensions were drawn into 1-ml syringes connected to polymer tubing, which was attached to a microfluidic chip composed of cured polydimethylsiloxane bonded to a cover glass (Flic20, Zellmechanik Dresden, Germany). At a sample flow rate of 0.03 µl/s and a sheath flow of 0.09 µl/s, cells were acquired within the channel area of the chip at a square measurement channel cross section of 20 µm × 20 µm. Cells were detected in a region of interest of 250 × 80 pixels at a frame rate of 2,000 frames per second using an image analysis algorithm that detects the contour of each cell, and its convex hull, in real time (19). A minimum of 5,000 events were collected and analysis of the convex hull contour of the cells performed using the open source software ShapeOut available on GitHub (https://github.com/ZELLMECHANIK-DRESDEN/ShapeOut). The ratio of convex hull area to cell contour area was defined as the area ratio, a novel and very sensitive surrogate quantifying shape changes as defined by Toepfner and colleagues (21).

### Glycogen Assay

The glycogen content of neutrophils, eosinophils, and HepG2 cells was determined using a glycogen assay kit (Abcam, UK) and normalized to protein content (BCA, Bio-Rad, UK).

### Statistical Analyses

All data represent the mean (±SEM) of (*n*) separate experiments unless otherwise stated. Statistical analyses were performed using GraphPad Prism (6.0d, San Diego, CA, USA) as detailed in the figure legends. Data were tested for normality using the Shapiro–Wilk test. For non-parametric data a Mann–Whitney *U* test was applied and for parametric data a one-way ANOVA (with Tukey *post hoc* test) or Student’s *t*-test was used. *P* < 0.05 was considered significant.

## Results

### Optimization of Extracellular Flux Measurement in Eosinophils

**Figure 1:**
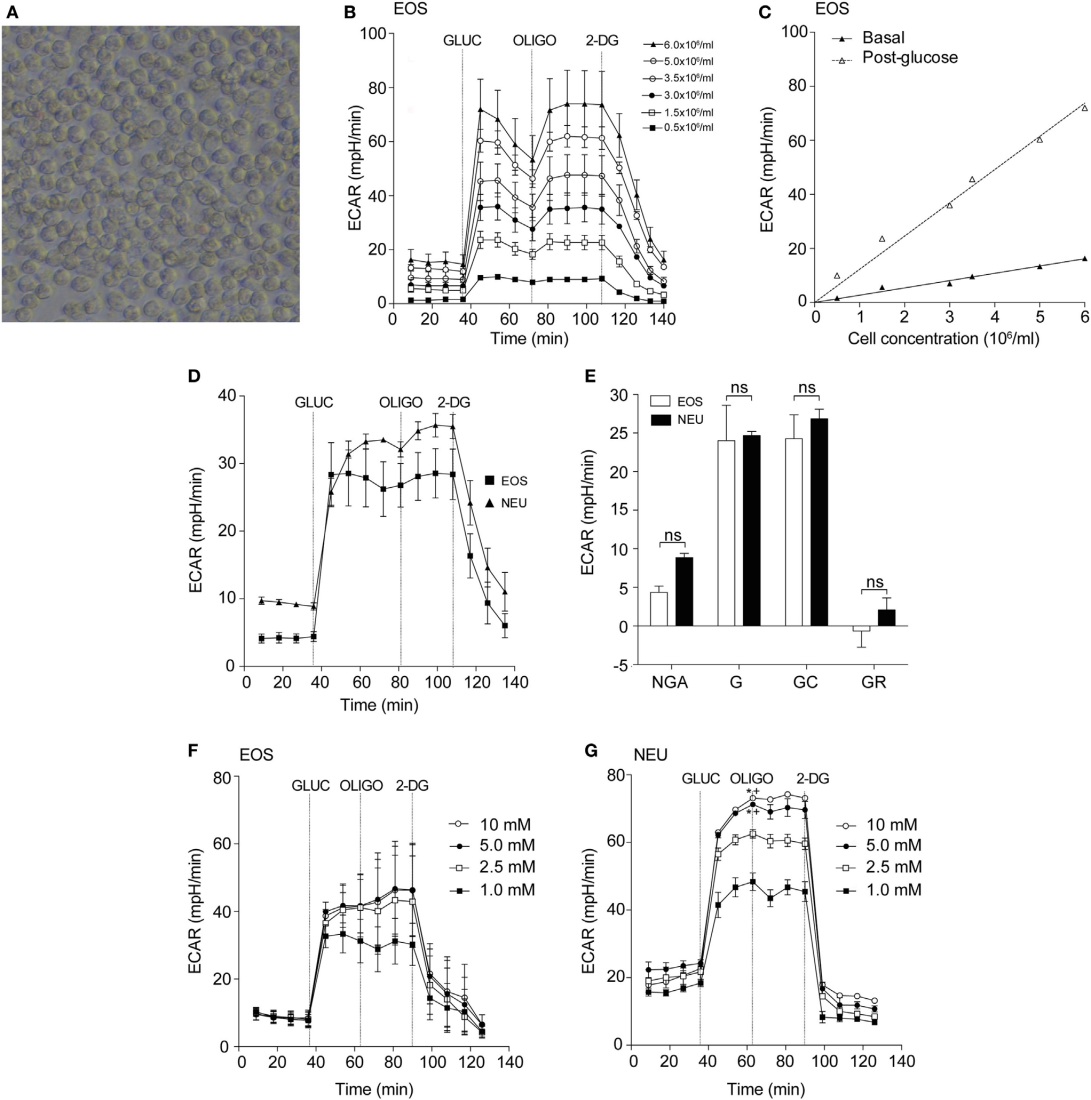
Optimization of eosinophil seeding density and measurement of glycolysis in eosinophils and neutrophils. **(A)** Representative light microscopy of eosinophils (3 × 10^6^/ml) adhered to a Cell-Tak-coated 96-well plate for 1 h. Magnification 40×. **(B)** Kinetic extracellular acidification rate (ECAR) response of eosinophils to glucose injection at 36 min (GLUC, 10 mM), the mitochondrial ATP synthase inhibitor oligomycin (OLIGO) injection at 72 min (OLIGO, 2.5 µM), and the inhibitor of glucose 2-DG (100 mM) injection at 108 min, using 0.5–6 × 10^6^ eosinophils/ml. Data represent the mean of a single experiment with ± SD of triplicate wells. **(C)** Eosinophil seeding density titration using basal and post-glucose ECAR levels. Data represent the mean ± SD of a single experiment. **(D)** Kinetic ECAR response of eosinophils (black squares) and neutrophils (black triangles) to stimulation with glucose (GLUC) at 36 min, OLIGO at 81 min, and 2-DG at 108 min. Data represent the mean ± SEM of three independent experiments. Eosinophil and neutrophil ECAR responses were measured simultaneously on the same assay plate. **(E)** Measurement of glycolytic rates in eosinophils (white bars) and neutrophils (black bars). Non-glycolytic acidification (NGA), glycolysis (G), glycolytic capacity (GC), and glycolytic reserve (GR). Data represent the mean ± SEM of three independent experiments, analyzed using one-way ANOVA test with Tukey *post hoc* test. Ns indicates a non-significant difference. **(F)** Eosinophil ECAR following stimulation with glucose (GLUC) (1–10 mM) at 36 min, OLIGO (2.5 µM) at 72 min, and 2-DG (100 mM) at 90 min. Data represent the mean ± SEM of three independent experiments, analyzed using one-way ANOVA with Tukey *post hoc* test. **(G)** Neutrophil ECAR following stimulation with glucose (GLUC) (1–10 mM) at 36 min, OLIGO (2.5 µM) at 72 min, and 2-DG (100 mM) at 90 min. Data represent the mean ± SEM of three independent experiments, analyzed using one-way ANOVA test with Tukey *post hoc* test. *p < 0.05 compared with 1 mM glucose and ^+^*p* < 0.05 compared with 2.5 mM glucose. Eosinophil and neutrophil ECAR responses were measured simultaneously on the same assay plate **(F,G)**.

To determine the optimal conditions required to study purified blood eosinophils in an extracellular flux assay, we first determined the optimal cell concentration required for flux measurements (Figure 1A). As shown in Figure 1B, the glycolytic response of eosinophils was cell concentration-dependent with a linear relationship between cell concentration and basal or post-glucose ECAR (Figure 1C). After visual inspection of the adherent cells, a cell concentration of 3 × 10^6^/ml (or 150 × 10^3^ per well) was chosen as this resulted in an evenly distributed monolayer of eosinophils with no observable shape change (Figure 1A). Cell concentrations above 3 × 10^6^/ml were found to result in eosinophil clumping. A concentration of 3 × 10^6^/ml was also found to be optimal for neutrophil ECAR responses (data not shown) and this concentration was used for both cell types throughout the study. Given the well-described susceptibility of neutrophils to activation and that granulocytes are adherent to Cell-Tak throughout the flux assay, we assessed the contribution of Cell-Tak adherence to neutrophil function. We found that contact with Cell-Tak had surprisingly little impact on luminol-dependent chemiluminescence even in GM-CSF or TNF-α primed neutrophils (Figure S1 in Supplementary Material).

### Eosinophils and Neutrophils Display Similar Glycolytic Responses but Differing Oxygen Consumption Rates

Having established the optimal flux assay conditions, we then sought to compare the metabolic profile of eosinophils and neutrophils in terms of glycolysis and oxygen consumption. Using the flux analyser we demonstrate that eosinophils and neutrophils have similar ECAR responses in terms of glycolysis rates, GR, GC, and NGA (Figures 1D,E). To determine the influence of glucose concentration on ECAR responses, we tested different concentrations of glucose (1–10 mM) in the assay medium. The eosinophil ECAR response was not greatly influenced by external glucose levels as illustrated in Figure 1F; eosinophil post-glucose ECAR peak at 1 mM glucose is 31.2 ± 6.4 mpH/min at 1 mM glucose compared to 41.6 ± 7.9 mpH/min at 10 mM glucose. In contrast, neutrophils exhibited a concentration-dependent ECAR response to glucose (Figure 1G). The neutrophil post-glucose ECAR peak was 48.4 ± 2.6 mpH/min at 1 mM glucose compared to 73.1 ± 0.4 mpH/min at 10 mM glucose (*p* < 0.05). Given the distinctive reliance of neutrophils on glycolysis we also compared the abundance of glycogen stores between neutrophils and eosinophils. Figures S2A,B in Supplementary Material illustrates that freshly isolated neutrophils have ~3-fold higher glycogen stores compared to eosinophils, a value significantly higher than HepG2 cells.

**Figure 2:**
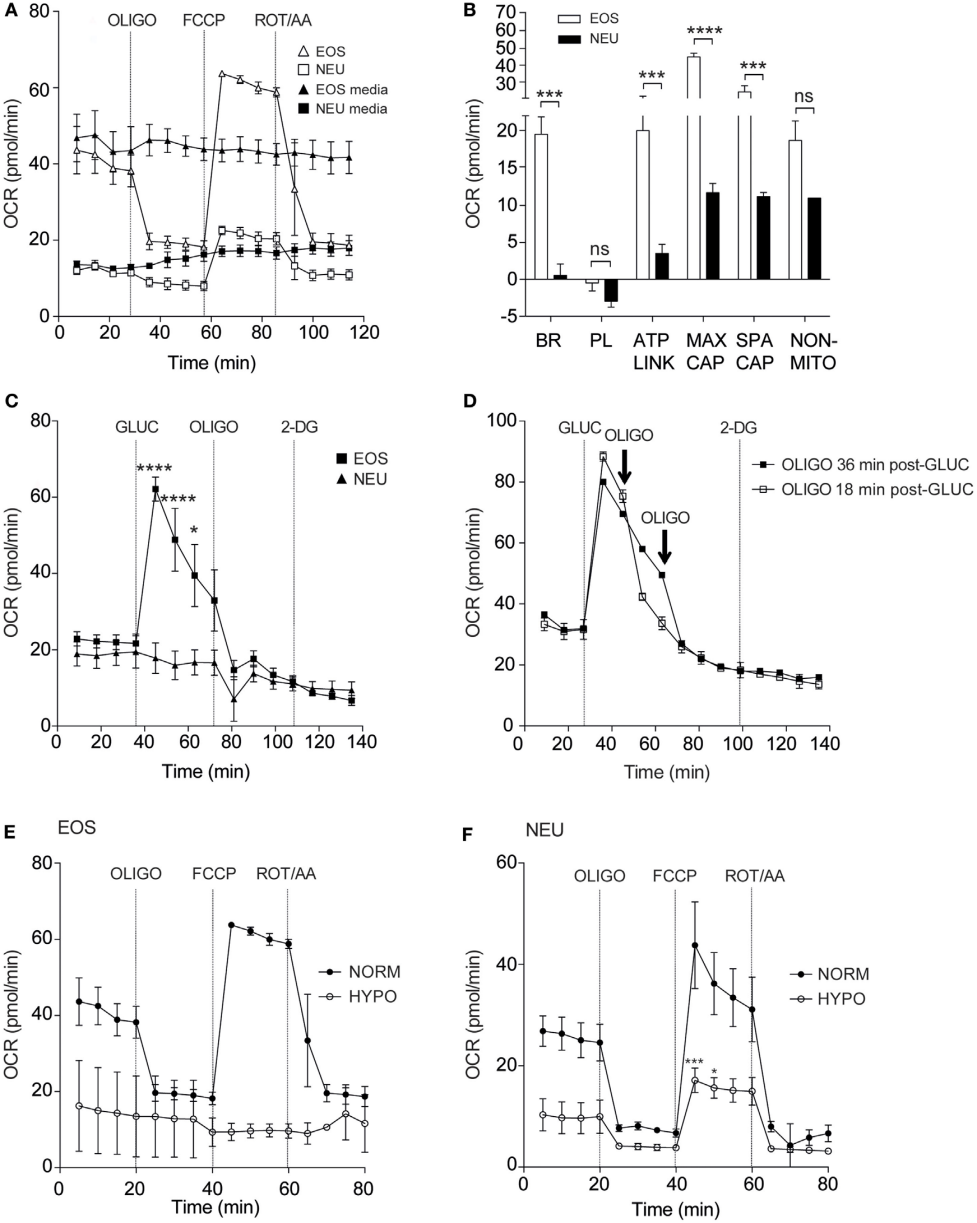
Eosinophil and neutrophil oxygen consumption rates under normoxia and hypoxia. **(A)** Kinetic oxygen consumption rates (OCR) of eosinophils (open triangles) and neutrophils (open squares) following injection of oligomycin (OLIGO) at 28 min, FCCP at 57 min and rotenone with antimycin A (mitochondrial complex I and III inhibitors, respectively) (ROT/AA) at 86 min. Closed triangles and closed squares represent the media only injections for eosinophils and neutrophils, respectively. Data represent the mean ± SEM of three independent experiments. **(B)** Comparison of oxidative parameters in eosinophils (white bars) and neutrophils (black bars). Basal respiration (BR), proton leak (PL), ATP-linked production (ATP LINK), maximum respiration capacity (MAX CAP), spare respiratory capacity (SPA CAP), and non-mitochondrial-derived OCR (NON-MITO). Eosinophil and neutrophil OCR responses were measured simultaneously on the same assay plate. Data represent the mean ± SEM of three independent experiments, ***p < 0.001 and ****p < 0.0001 compared with equivalent neutrophil values (one-way ANOVA test with Tukey *post hoc* test). Ns indicates a non-significant difference. **(C)** Measurement of OCR in eosinophils (closed squares) and neutrophils (closed triangles) in response to glucose (GLUC) injection at 36 min, OLIGO injection at 73 min and 2-DG injection at 108 min. Eosinophil and neutrophil OCR responses were measured simultaneously on the same assay plate. Data represent the mean ± SEM of three independent experiments. *p < 0.05 and ****p < 0.0001 compared with equivalent neutrophil values (one-way ANOVA test with Tukey *post hoc* test). **(D)** Eosinophil OCR in response to the addition of OLIGO 18 (open squares) or 36 min (closed squares) after GLUC injection. Data represent the mean ± SD of a single representative experiment (representative of 3). **(E)** Eosinophil OCR under normoxia (NORM) or hypoxia (HYPO) in response to stimulation by OLIGO at 20 min, FCCP at 40 min, and rotenone with antimycin A (ROT/AA) injection at 60 min. Data represent the mean ± SEM of three independent experiments under normoxia and a single independent experiment under hypoxia (mean ± SD). **(F)** Neutrophil OCR under normoxia (NORM) or hypoxia (HYPO) in response to stimulation with OLIGO at 20 min, FCCP injection at 40 min, and rotenone with antimycin A (ROT/AA) injection at 60 min. Data represent the mean ± SEM of ≥3 independent experiments. ***p < 0.001 and *p < 0.05 compared with normoxia 45 and 50 min values (one-way ANOVA test with Tukey *post hoc* test). Eosinophil and neutrophil responses were measured simultaneously on the same assay plate **(E,F)**.

Figure 2A shows the real-time OCR responses of eosinophils and neutrophils using reagents that disrupt the mitochondrial respiratory chain such as the uncoupling agent FCCP, the mitochondrial ATP synthase inhibitor OLIGO and Rot/AA (mitochondrial complex I and III inhibitors, respectively). When these responses were compared between the cell types, we observed that basal respiration, ATP-linked respiration, maximum respiratory capacity, and spare respiratory capacity were all significantly increased in eosinophils compared to neutrophils (Figure 2B). Of note, eosinophil OCR increased significantly from 22.9 ± 1.9 pmol/min at baseline to 62.1 ± 3.2 pmol/min (*p* < 0.0001, Figure 2C) following the addition of glucose, whereas neutrophil OCR remained unchanged. This response is attributable to eosinophil glucose oxidation as the levels of OCR decreased substantially following the early (18 min post-glucose) injection of OLIGO (Figure 2D). Given that our assay media contained glutamine, we also examined whether eosinophils could undergo glutamine oxidation (17). However, as shown in Figure S3A in Supplementary Material, addition of 2 mM glutamine to eosinophils in glucose-free and glutamine-free media had no impact on OCR or ECAR, suggesting this is not a major energy pathway. Similarly, neutrophil OCR and ECAR remained unchanged upon injection of glutamine (Figure S3B in Supplementary Material).

It is well known that hypoxia can influence metabolism in a wide variety of cell types (22, 23). We, therefore, examined eosinophil and neutrophil OCR responses under hypoxia (3 kPa; 0.8% O_2_, and 0.1% CO_2_). Granulocytes were exposed to hypoxia for 2 h prior to assessment of mitochondrial respiration. Addition of FCCP increased eosinophil OCR under normoxia (63.8 ± 0.7 pmol/min) but this response was reduced to 9.4 ± 1.6 pmol/min under hypoxia (Figure 2E). Although less pronounced, neutrophil OCR was also reduced as shown in Figure 2F. The post-FCCP neutrophil OCR decreased from 43.8 ± 8.5 pmol/min during normoxia to 17.1 ± 2.4 pmol/min (*p* < 0.001) under hypoxia.

### Priming Agents Increase Eosinophil Oxygen Consumption Rates

**Figure 3:**
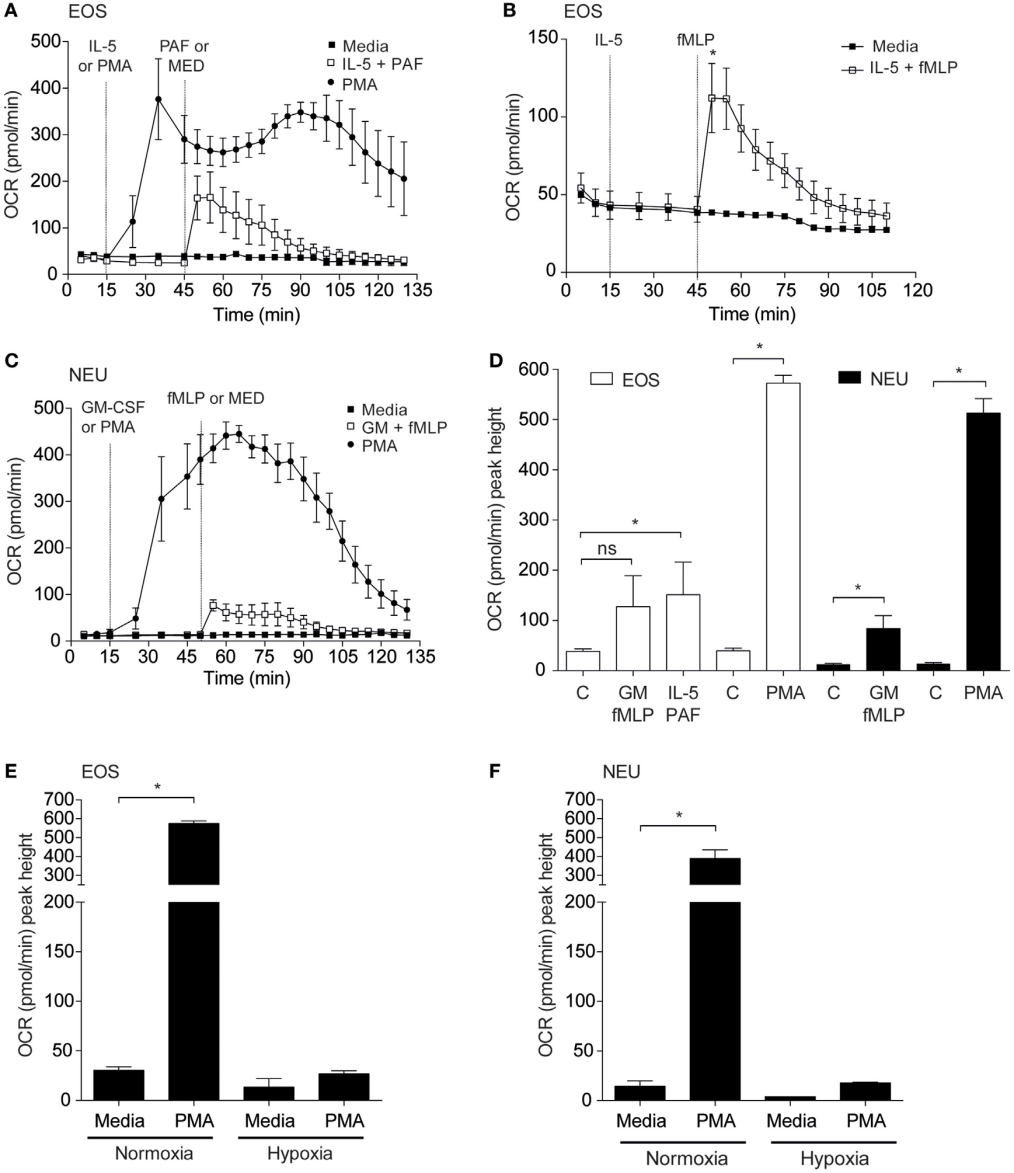
Effect of priming agents on eosinophil and neutrophil oxygen consumption rates. **(A)** Kinetic oxygen consumption rates (OCR) of eosinophils following injection of PMA (closed circles, 200 nM) at 15 min or PAF (open squares, 5 µM) at 45 min. Eosinophils stimulated with PAF were exposed to IL-5 (10 ng/ml) for 30 min prior to injection of PAF. Closed squares represent the media only injections. Data represent the mean ± SEM of three independent experiments. **(B)** Kinetic oxygen consumption rates (OCR) of eosinophils following injection of IL-5 alone at 15 min followed by fMLP injection (open squares, 100 nM) at 45 min. Closed squares represent the media only injections. Data represent the mean ± SEM of seven independent experiments (*p < 0.05 compared with media alone using a Student’s *t*-test). **(C)** Kinetic OCR responses of neutrophils following injection of PMA (closed circles, 200 nM) at 15 min or fMLP (open squares, 100 nM) at 50 min. Neutrophils stimulated with fMLP were injected with GM-CSF (10 ng/ml) for 35 min prior to fMLP treatment. Black squares represent the media only injections. Data represent the mean ± SEM of three independent experiments. **(D)** Comparison of the peak height OCR by eosinophils (white bars) and peak height OCR by neutrophils (black bars). Treatments were performed as described in **(A,C)**. Data represent the mean ± SEM of four independent experiments for GM-CSF/fMLP stimulation, five independent experiments for PAF/fMLP, and ≥4 independent experiments for PMA (*p < 0.05 compared with media alone using a Student’s *t*-test). Ns indicates a non-significant difference. **(E)** Comparison of eosinophil peak height OCR following PMA (200 nM) injection under normoxia or hypoxia. Data represent the mean ± SEM of ≥4 independent experiments under normoxia (*p < 0.05 compared with media alone using a Student’s *t*-test) and mean ± SD of two independent experiments under hypoxia. **(F)** Comparison of neutrophil peak height OCR following PMA (200 nM) injection under normoxia or hypoxia. Data represent the mean ± SEM of ≥4 independent experiments under normoxia (*p < 0.05 compared with media alone using a Student’s *t*-test) and mean ± SD of two independent experiments under hypoxia. Eosinophil and neutrophil OCR responses were measured simultaneously on the same assay plate **(D,E)**.

Granulocyte priming by agents such as GM-CSF or IL-5 are known to markedly enhance agonist-stimulated respiratory burst (24, 25). We sought to investigate the effect of these priming agents on eosinophil and neutrophil oxygen consumption rates. Eosinophils incubated inside the flux analyser were stimulated with IL-5 for 15 min before addition of PAF or fMLP and OCR measurements acquired (Figures 3A,B). Eosinophils exhibited enhanced OCR from 38.5 ± 4.8 pmol/min to 150.9 ± 65.1 pmol/min when primed with IL-5 and stimulated with PAF (*p* < 0.05). The PKC activator PMA also increased OCR by eosinophils from 24.5 ± 5.4 to 572.8 ± 15.8 pmol/min (*p* < 0.0001) as measured by the peak height OCR. Similarly, eosinophil OCR increased from 38.6 ± 6.8 to 112.1 ± 22.3 pmol/min when primed with IL-5 and stimulated with fMLP (*p* < 0.05). Injection of IL-5 alone had no effect on baseline OCR or ECAR (Figure S4 in Supplementary Material). As shown in Figure 3C GM-CSF primed neutrophils stimulated with fMLP also show an increase in OCR from 12.5 ± 1.9 to 84.3 ± 25.5 pmol/min in GM-CSF-primed neutrophils (*p* < 0.05). Neutrophils exhibited an uplift in OCR when stimulated with PMA (from 13.9 ± 2.2 to 513 ± 28.8 pmol/min with PMA (*p* < 0.0001)). Notably however, the kinetics of the eosinophil and neutrophil OCR response to PMA differed markedly; neutrophil OCR returned close to baseline levels after 135 min, whereas eosinophils displayed a biphasic and more sustained OCR over the equivalent time period. Figure 3D summarizes the peak height oxygen consumption rates in eosinophils and neutrophils in response to PAF, fMLP, and PMA.

We next evaluated the influence of hypoxia on eosinophil and neutrophil OCR in response to PMA treatment (Figures 3E,F). As anticipated, basal and PMA-induced OCR in eosinophils was profoundly attenuated under hypoxic conditions (27.0 ± 3.0 pmol/min under hypoxia compared to 574.4 ± 14.2 pmol/min under normoxia). In a similar manner neutrophil OCR responses to PMA were also reduced by approximately 95% under hypoxia (389.7 ± 32.3 vs 17.8 ± 0.8 pmol/min) (Figure 3F).

### Eosinophil Morpho-Rheological Profile and Oxygen Consumption Rates in Atopic and Non-Atopic Subjects

**Figure 4:**
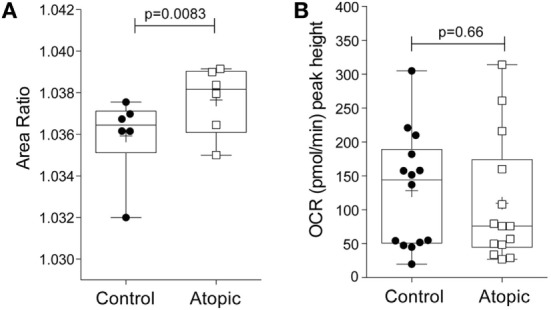
Morpho-rheological properties and oxygen consumption rates in eosinophils from atopic and non-atopic subjects. **(A)** RT-DC was performed on freshly isolated eosinophils from non-atopic volunteers (controls) and atopic volunteers to calculate the area ratio, as described in Section “Materials and Methods.” Box-and-whiskers plot shows median ± range of six independent experiments with mean shown as +, *p < 0.05 compared with non-atopic controls (Student’s *t* test). **(B)** Comparison of the peak height OCR by eosinophils from non-atopic and atopic subjects primed with IL-5 (10 ng/ml) for 15 min and then stimulated with fMLP (100 nM). Box-and-whiskers plot shows median ± range of 14 independent experiments with mean shown as +, *p < 0.05 compared with non-atopic controls (Mann–Whitney test).

To determine whether the morpho-rheological and metabolic properties of eosinophils are altered in atopic conditions, eosinophils were isolated from atopic subjects and compared to eosinophils from non-atopic subjects. We first compared the morpho-rheological properties of atopic and non-atopic eosinophils using RT-DC (detailed in Figures S5A–C in Supplementary Material). The measurement of total area ratio by RT-DC acts as a sensitive, label-free, marker of granulocyte shape change (21). Figure 4A shows that eosinophils from atopic subjects display a significant increase in the total area ratio compared to eosinophils from non-atopic subjects (*p* = 0.008), comparable with basal priming. Additional morpho-rheological parameters were also assessed, including eosinophil deformability and area, but these parameters were unchanged between the atopic and non-atopic eosinophils (Figures S5D,E in Supplementary Material). When the peak height OCR was measured (Figure 4B), we found that there was no difference between IL-5-primed and fMLP-stimulated eosinophils from atopic subjects compared to non-atopic eosinophils (*p* = 0.66).

## Discussion

Human neutrophils are known to be primarily glycolytic cells, harnessing aerobic glycolysis to perform their critical functions (11, 12). While a lot is known about the dependence of neutrophils on glycolysis, the metabolic characteristics of eosinophils have been less well defined. Recent technological advances have enabled the real-time assessment of cellular bioenergetics in intact primary cells (14, 26). These improvements avoid many of the artifacts previously associated with mitochondrial isolation or cell permeabilization and thus have far greater physiological relevance. In this study, we have used a real-time extracellular flux analyser to characterize for the first time the metabolic profile of human blood-derived eosinophils and compared directly their profile to neutrophils.

Eosinophils demonstrated a number of bioenergetic differences compared to neutrophils. First, they consume more oxygen than neutrophils as demonstrated by their enhanced basal rate of OCR, ATP-linked OCR, maximal capacity, and spare capacity. These data are consistent with previous studies, which show that neutrophils are primarily glycolytic, and have fewer mitochondria than other circulating leukocytes (27). In fact, Peachman and colleagues have calculated that there are only 5–6 mitochondria per neutrophil, while a similar quantification in eosinophils has identified 24–36 mitochondria per eosinophil (28). Second, our studies reveal that eosinophils can utilize glucose oxidation as an alternative energy pathway. By undergoing glucose oxidation, glucose-derived pyruvate can enter the mitochondria and thus participate in the tricarboxylic acid cycle. Previous work has shown that eosinophils display enhanced glucose oxidation compared to neutrophils as measured by oxidation of ^14^C-glucose (29). However, these results were somewhat provisional as they relied on eosinophils derived from a single patient with eosinophilia. Glutamine oxidation has been observed in rat neutrophils (30); however, in our experiments, human eosinophil and neutrophil OCR and ECAR were unchanged in response to injection of glutamine.

Our study demonstrates that eosinophils, like neutrophils, also utilize glycolysis as an energy pathway. Although not directly indicative of active glycolysis, Venge and colleagues have shown uptake of 18-FDG by human eosinophils and describe the involvement of the glucose transporter molecules (GLUTs) 1, 3, and 4 in this process (31). While neutrophils and eosinophils both undergo glycolysis, we noted that eosinophils were less responsive to increasing concentrations of glucose compared to neutrophils, perhaps due to the reliance of neutrophils on glycolysis. Future metabolomics analysis and/or glucose uptake assays will help further determine the glucose dependency of eosinophils compared to neutrophils. Of interest, neutrophils display a threefold higher glycogen content compared to eosinophils, and this finding may reflect the additional ability of neutrophils to utilize glycogen stores for energy production (glycogenolysis). Moreover, Robinson and colleagues have shown that guinea-pig neutrophils can increase their glycogen content as they leave the circulation and enter sites of inflammation (32).

Environmental signals, such as cytokines, growth factors, and oxygen availability, can all contribute to metabolic regulation. In the case of eosinophils the cytokine IL-5 enhances eosinophil activation and differentiation, and acts as an anti-apoptotic (pro-survival) signal (33). Moreover, eosinophils obtained from the bronchial lavage of atopic asthmatics show increased expression of IL-5 mRNA following allergen challenge (34) and there is a correlation between the IL-5 concentration in the serum and severity of asthma (35). Our data show that IL-5 primed and fMLP-stimulated eosinophils undergo a profound increase in their OCR. This response highlights how the inflammatory milieu can alter the metabolic profile of eosinophils, and conversely, how these cells might contribute to local tissue hypoxia.

Previous studies have demonstrated that, like neutrophils, stimulated eosinophils can undergo a respiratory burst (36, 37). The highly reactive oxygen metabolites thus generated perform a protective function in both granulocytes; principally bactericidal in neutrophils and anti-parasitic in eosinophils. Petreccia and colleagues (36) showed that relative to neutrophils, eosinophils displayed higher levels of superoxide anion production both at rest and following PMA stimulation. Consistent with these findings, not only did eosinophils have a higher basal OCR but also PMA stimulation promoted a far more sustained increase in oxygen consumption in eosinophils relative to neutrophils, although the maximal OCRs achieved was not too dissimilar.

Inflamed lesions are often severely hypoxic due to increased cellular oxygen demand and reduced oxygen availability (38–40). Hypoxia is a potent pro-inflammatory stimulus for both neutrophils and eosinophils, contributing to an anti-apoptotic and a hyper-secretory profile in both cell types (notably enhanced release of neutrophil elastase and myeloperoxidase from neutrophils and Charcot–Leyden crystal/Galectin-10 production by eosinophils) (22, 40). In our study, hypoxia profoundly suppressed mitochondrial oxidative phosphorylation in eosinophils, indicative of a metabolic pathway shift from oxidative phosphorylation to glycolysis. This finding is supported by our previously published data, showing upregulation of GLUT1 in eosinophils under hypoxic conditions (40). Such metabolic changes are likely attributable to the activation of hypoxia-inducible factor-1-alpha (HIF-1α) signaling, which is known to increase the levels of glycolytic enzymes and glucose transporters through HIF-1α responsive elements, while downregulating mitochondrial metabolism (39). This shift to glycolysis may allow eosinophils to operate in an inflammatory environment where the oxygen availability is restricted.

Our study had certain limitations. First, we focused on glucose and glutamine as the primary substrates and did not explore extensively alternative energy sources such as fatty acids and alanine. Few studies have investigated the role of these alternative substrates in granulocyte biology but a role for fatty acid metabolism in neutrophil differentiation has been recently identified (41). Second, we only considered blood-derived eosinophils and the metabolic profile of tissue-resident eosinophils may differ. Mesnil and colleagues have identified lung resident eosinophils (rEos) that are distinguished from “influxing” inflammatory eosinophils (iEos) by their higher CD62L expression, low CD101 expression, and notable expression of immunoregulatory genes (8).

Eosinophils obtained from atopic donors are known to be functionally distinct from cells in non-atopic donors, demonstrating enhanced respiratory burst and a greater proportion of hypodense granules (25, 42). In agreement with these data, we find alterations in the shape or external contour of atopic eosinophils compared to non-atopic eosinophils. Changes in eosinophil shape may occur because eosinophils from atopic volunteers are thought to be primed or partially primed *in vivo* (43). Eosinophils undergo shape change *in vitro* in response to agonists, such as IL-5, eotaxin, and platelet-activating factor (44, 45), but differences in baseline shape change have not been previously demonstrated between atopic and non-atopic eosinophils. Given these attributes, we predicted that eosinophils from atopic donors would be more metabolically active. However, we found no significant difference in the oxygen consumption between atopic and non-atopic eosinophils following treatment with IL-5 and fMLP. Whether there are differences in the basal glycolytic rate or oxidative phosphorylation levels between atopic and non-atopic eosinophils remains to be investigated. Intriguingly, a study comparing the transcriptional profile of circulating eosinophils in asthmatics and healthy subjects found that acyl-CoA thioesterase 4 was upregulated in asthmatics (46). It is, therefore, tempting to speculate that alterations in fatty acid metabolism may contribute to the phenotypic changes exhibited by eosinophils from asthmatics (47).

Overall, our data indicate that eosinophils are more metabolically “flexible” than neutrophils, and that eosinophils can adapt more readily under conditions of altered energy demands. This flexibility may allow the eosinophil to operate not only as a cytotoxic effector cell but also as a participant in immune homeostasis.

## Ethics Statement

This study was approved by the Cambridgeshire 2 Research Ethics Committee (06/Q0108/281). All subjects gave written informed consent in accordance with the Declaration of Helsinki.

## Author Contributions

EC, NF, and MA conceived and designed the experiments. LP, NF, NT, and KB performed the experiments and analyzed the data. NF wrote the manuscript with input from LP, NT, KB, JG, MA, and EC. All authors contributed to the drafting and revising of the manuscript and have approved the final version.

## Conflict of Interest Statement

The authors declare that the research was conducted in the absence of any commercial or financial relationships that could be construed as a potential conflict of interest.

## Supplementary Material

Figure S1 Effect of Cell-Tak-coated wells on reactive oxygen species generation by neutrophils. Freshly isolated neutrophils were left to adhere onto Cell-Tak coated wells for 5 min after priming in suspension (control) or primed while adhered to Cell-Tak coated wells (pre-prime + Cell Tak). Data represent the mean ± SD of two independent experiments.

Figure S2 Glycogen content of freshly isolated eosinophils, neutrophils, and HepG2 cells. **(A)** Glycogen content (pg/cell) was measured by enzymatic assay as described in the Section “Materials and Methods.” Data represent the mean ± SEM of ≥3 independent experiments. **p* < 0.05 compared with HepG2 samples (one-way ANOVA with Tukey *post hoc* test). **(B)** Glycogen content expressed per milligram protein concentration. Data represent the mean ± SD of ≥2 independent experiments. Ns indicates a non-significant difference.

Figure S3 Kinetic extracellular acidification rate (ECAR) and OCR response of eosinophils and neutrophils to glutamine stimulation. **(A)** Eosinophil ECAR and OCR response to glutamine injection (GLUT, 2 mM) at 36 min, oligomycin (OLIGO, 2.5 µM) at 72 min and 2-DG (100 mM) at 108 min. **(B)** Neutrophil ECAR and OCR response to glutamine injection (GLUT, 2 mM) at 36 min, OLIGO (2.5 µM) at 72 min, and 2-DG (100 mM) at 108 min. Data represent the mean ± SD of a single experiment, representative of two. Eosinophil and neutrophil ECAR and OCR responses were measured simultaneously on the same assay plate.

Figure S4 Kinetic extracellular acidification rate (ECAR) and OCR response of eosinophils to IL-5 stimulation. Eosinophil OCR and ECAR response to IL-5 injection (10 ng/ml) at 20 min, glucose injection (GLUC, 10 mM) at 35 min, oligomycin (OLIGO, 2.5 µM) at 60 min, and 2-DG (100 mM) at 75 min. Data represent the mean ± SD of two independent experiments.

Figure S5 Analysis of eosinophil cross-sectional area, deformation, and area ratio using RT-DC. Analysis was performed on freshly isolated eosinophils as described in Section “Materials and Methods.” **(A)** Within a constriction channel, shear stress and pressure act to deform the eosinophil. **(B)** An image analysis algorithm determines the contour of individual eosinophils (left) and its convex hull (right), and quantifies deformation and area ratio in real-time. The cross-sectional area is defined *via* cell contour detection as shown and convex cell contour and convex hull area are calculated. **(C)** Analysis reveals cell deformation and area ratio. Eosinophils with area ratios between 1 and 1.05 are used for calculation of cellular deformation. **(D)** Comparison of eosinophil cross-sectional area in eosinophils from atopic and non-atopic subjects. Box-and-whiskers plot shows median ± range of six independent experiments with mean shown as +, *p* values calculated using a Student’s *t*-test. **(E)** Comparison of eosinophil deformation in eosinophils from atopic and non-atopic subjects. Box-and-whiskers plot shows median ± range of six independent experiments with mean shown as +, *p* values calculated using a Student’s *t*-test.
