# Supplementary figures and images for: Metabolic Profiling of Human Eosinophils
Source: Front Immunol. 2018 Jun 21;9:1404. doi: 10.3389/fimmu.2018.01404 (PMC6036296; doi:10.3389/fimmu.2018.01404)

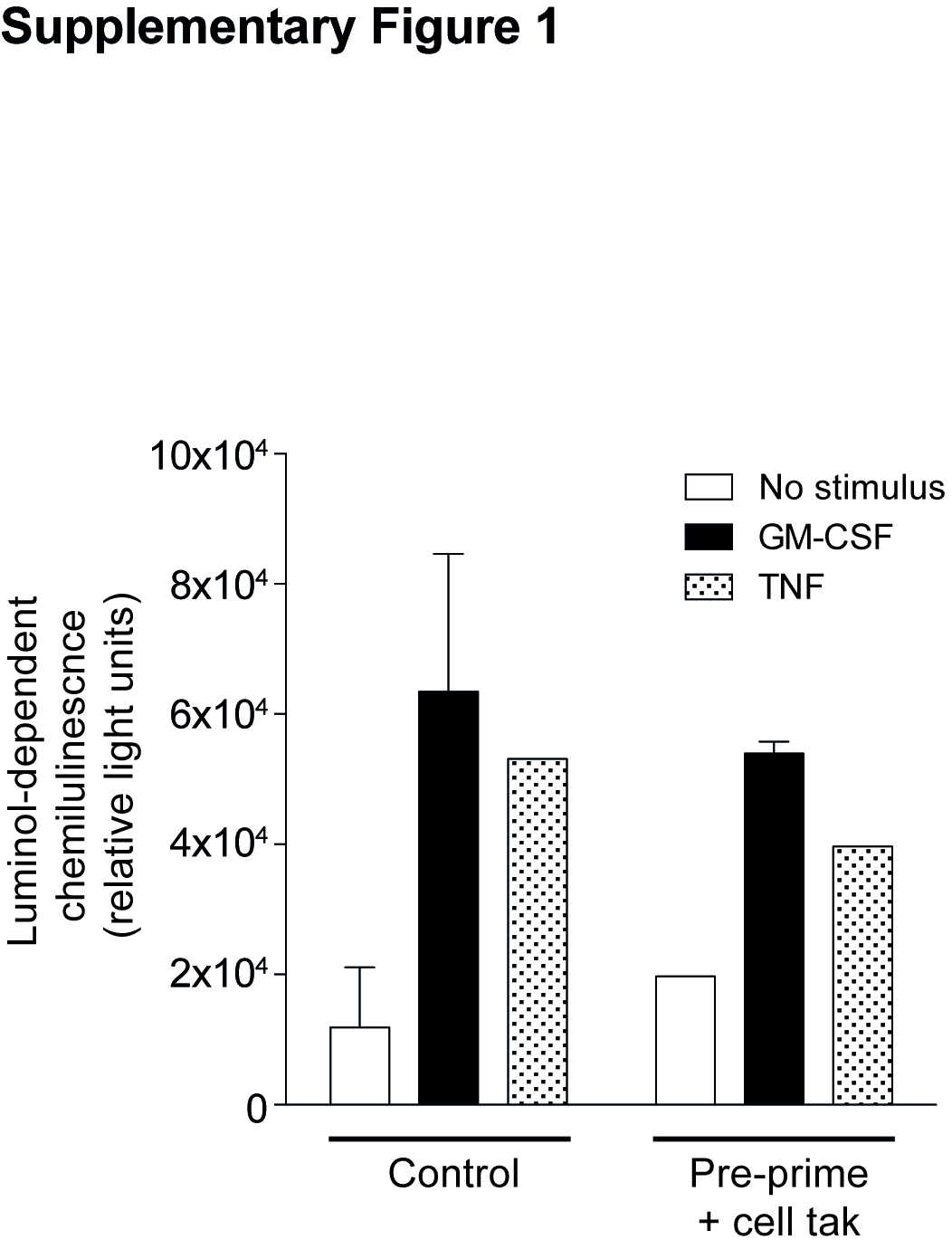

Supplement: Figure S1 — Effect of Cell-Tak-coated wells on reactive oxygen species generation by neutrophils. Freshly isolated neutrophils were left to adhere onto Cell-Tak coated wells for 5 min after priming in suspension (control) or primed while adhered to Cell-Tak coated wells (pre-prime + Cell Tak). Data represent the mean ± SD of two independent experiments. [file Image_1.tif]

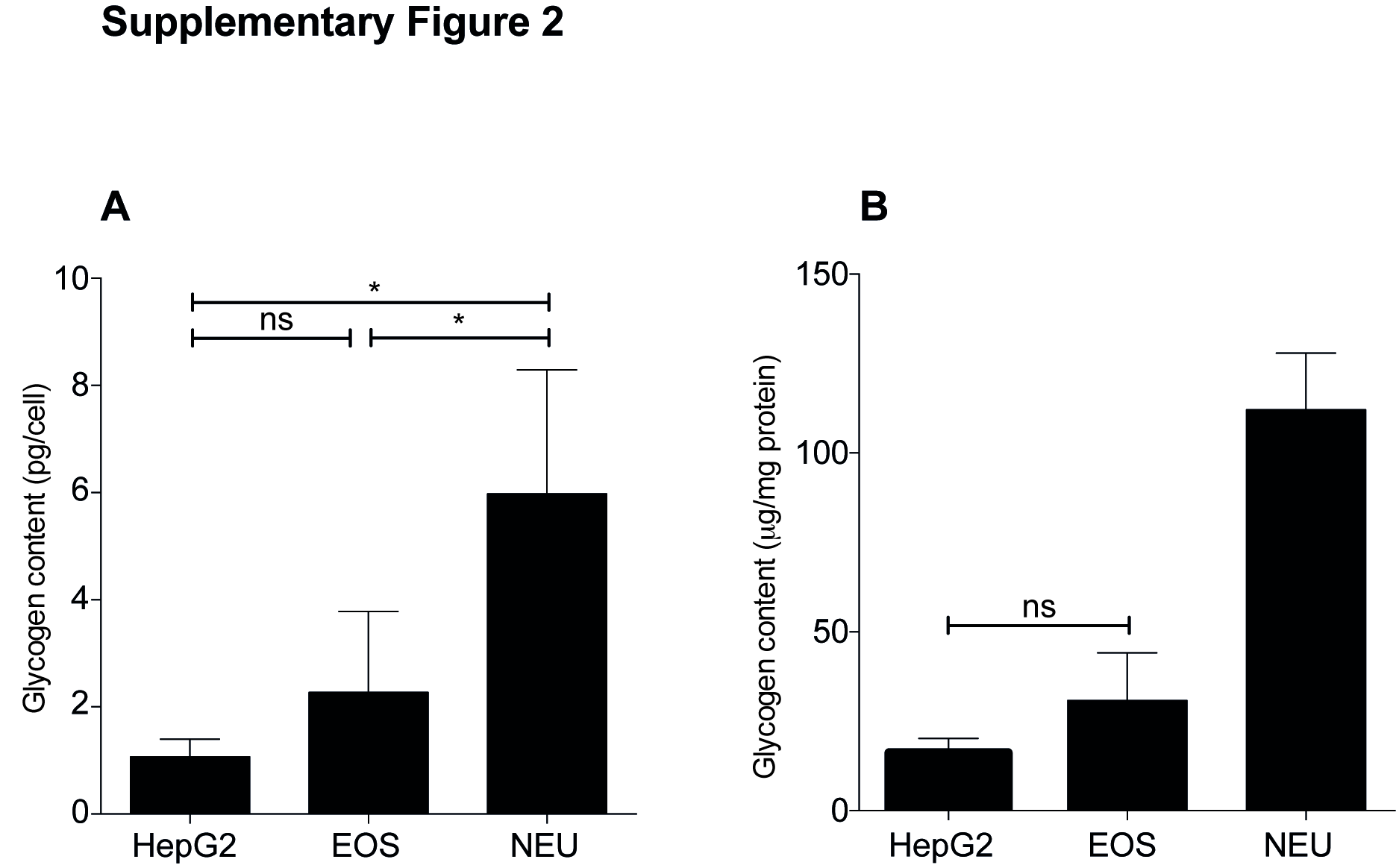

Supplement: Figure S2 — Glycogen content of freshly isolated eosinophils, neutrophils, and HepG2 cells. (A) Glycogen content (pg/cell) was measured by enzymatic assay as described in the Section “Materials and Methods.” Data represent the mean ± SEM of ≥3 independent experiments. *p < 0.05 compared with HepG2 samples (one-way ANOVA with Tukey post hoc test). (B) Glycogen content expressed per milligram protein concentration. Data represent the mean ± SD of ≥2 independent experiments. Ns indicates a non-significant difference. [file Image_2.tif]

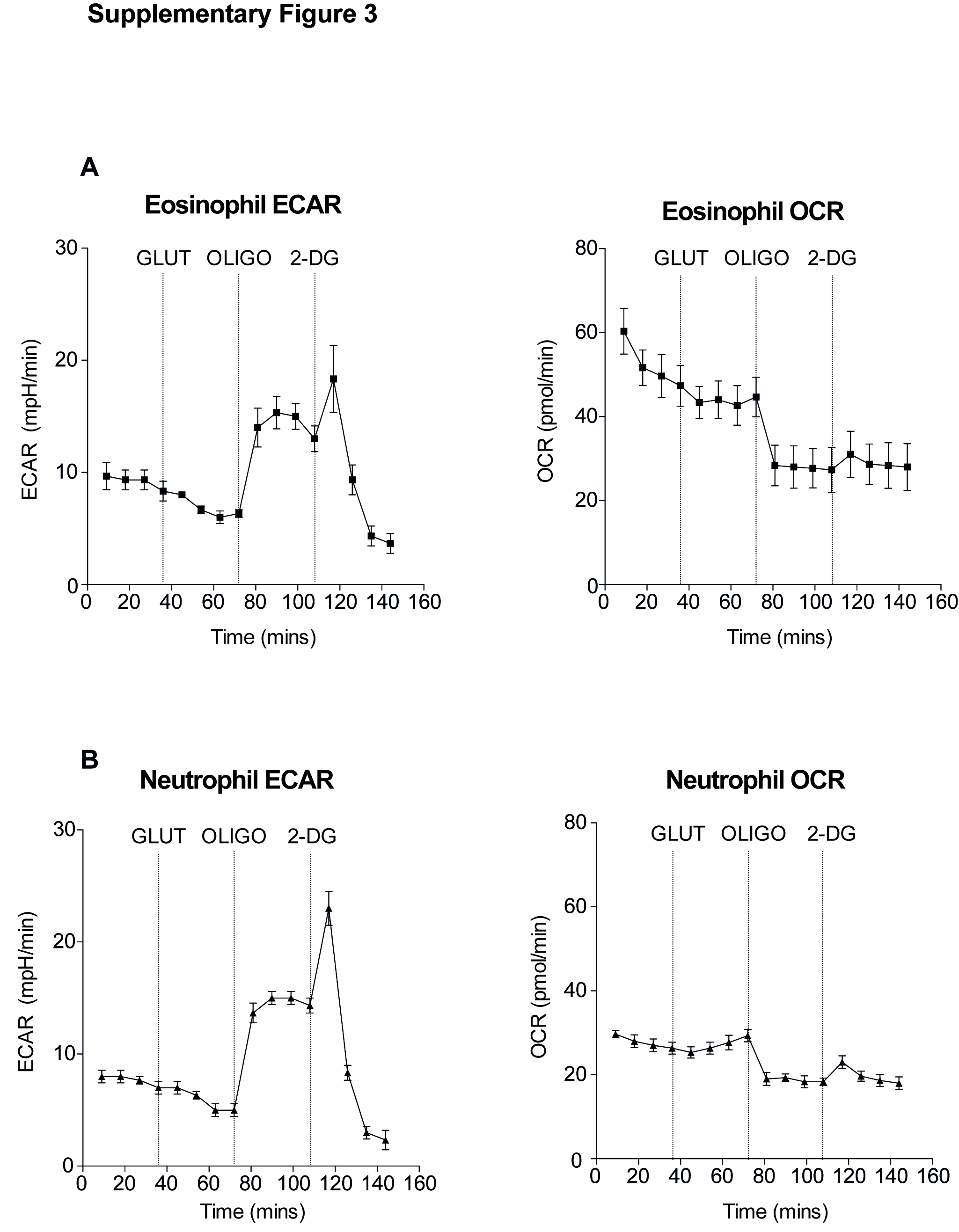

Supplement: Figure S3 — Kinetic extracellular acidification rate (ECAR) and OCR response of eosinophils and neutrophils to glutamine stimulation. (A) Eosinophil ECAR and OCR response to glutamine injection (GLUT, 2 mM) at 36 min, oligomycin (OLIGO, 2.5 µM) at 72 min and 2-DG (100 mM) at 108 min. (B) Neutrophil ECAR and OCR response to glutamine injection (GLUT, 2 mM) at 36 min, OLIGO (2.5 µM) at 72 min, and 2-DG (100 mM) at 108 min. Data represent the mean ± SD of a single experiment, representative of two. Eosinophil and neutrophil ECAR and OCR responses were measured simultaneously on the same assay plate. [file Image_3.tif]

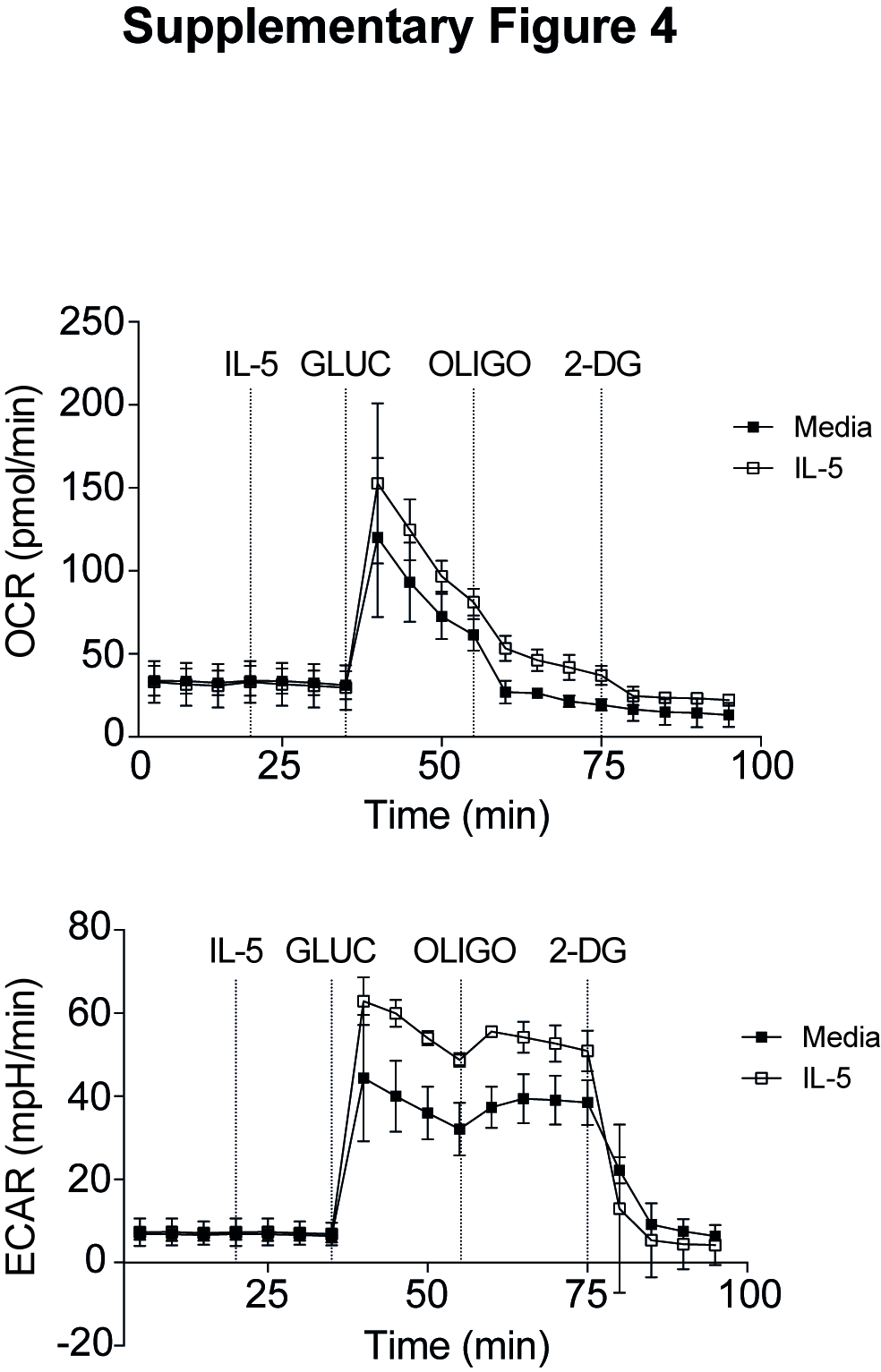

Supplement: Figure S4 — Kinetic extracellular acidification rate (ECAR) and OCR response of eosinophils to IL-5 stimulation. Eosinophil OCR and ECAR response to IL-5 injection (10 ng/ml) at 20 min, glucose injection (GLUC, 10 mM) at 35 min, oligomycin (OLIGO, 2.5 µM) at 60 min, and 2-DG (100 mM) at 75 min. Data represent the mean ± SD of two independent experiments. [file Image_4.tif]
